# Texture Analysis of Kidney MRI Using Diffusion-Weighted Imaging and Intravoxel Incoherent Motion for Classifying the Severity of Chronic Kidney Diseases

**DOI:** 10.7759/cureus.104887

**Published:** 2026-03-09

**Authors:** Hirokazu Shimizu, Keita Nagawa, Yuki Hara, Yuya Yamamoto, Tsutomu Inoue, Hirokazu Okada, Kaiji Inoue, Eito Kozawa

**Affiliations:** 1 Radiology, Saitama Medical University Hospital, Moroyama, JPN; 2 Radiology, National Hospital Organization Takasaki General Medical Center, Takasaki, JPN; 3 Nephrology, Saitama Medical University Hospital, Moroyama, JPN

**Keywords:** apparent diffusion coefficient, chronic kidney disease, diffusion‑weighted imaging, intravoxel incoherent motion, machine learning, magnetic resonance imaging, non-invasive assessment, renal dysfunction severity, texture analysis

## Abstract

Introduction

Chronic kidney disease (CKD) is a significant global health concern, and noninvasive imaging biomarkers for chronic kidney disease have been investigated using magnetic resonance imaging (MRI), including diffusion‑weighted imaging (DWI), blood oxygenation level-dependent (BOLD) imaging, arterial spin labeling (ASL), and T1 mapping; however, there are currently no widely accepted imaging biomarkers for the non-invasive assessment of renal dysfunction severity. DWI and intravoxel incoherent motion (IVIM), when combined with texture analysis (TA), provide a promising approach for the non-invasive assessment of renal microstructure. This study investigated the utility of TA applied to DWI/IVIM-derived maps for assessing the severity of renal dysfunction.

Materials and methods

We retrospectively analyzed kidney MRI data from 68 patients with CKD who underwent DWI-IVIM. Data were categorized into three groups based on the estimated glomerular filtration rates (eGFRs). Two-dimensional segmentation was performed on the apparent diffusion coefficient (ADC), true diffusion coefficient, pseudo-perfusion diffusion coefficient, and perfusion fraction map. After extracting 93 texture features (TFs), we employed a sequential feature selection algorithm for feature selection. Multiclass classification models were developed using linear discriminant analysis, quadratic discriminant analysis (QDA), support vector machines, k-nearest neighbors, decision trees, and random forest classifiers. To evaluate model performance, we performed a 10‑fold cross‑validation 100 times and used the mean of the resulting performance measures as the performance estimate.

Results

TA models demonstrated acceptable discriminatory performance, with the highest performance achieved by the ADC map-based QDA model (area under the curve or AUC 0.851±0.010). The IVIM-derived parameters did not outperform the ADC-based models. TFs derived from the left kidney showed slightly better results than those derived from the right kidney. There were no significant differences between TFs derived from the renal medulla and the cortex.

Conclusion

Although the combination of DWI-IVIM and TA has the potential to evaluate renal impairment in patients with CKD, the TA model based on the ADC map achieved higher accuracy than the models based on other IVIM parameters. Consequently, the utility of TA using ADC maps was reaffirmed. Future research with larger cohorts and improved image quality is needed to further refine the non-invasive assessment of renal dysfunction.

## Introduction

Chronic kidney disease (CKD) is a major global health concern that not only predisposes patients to end-stage renal disease (ESRD) requiring renal replacement therapy [1] but also increases the risk of cerebrovascular and cardiovascular events [2,3]. In addition, declining renal function impairs immune competence and increases susceptibility to severe infections such as pneumonia [4]. Consequently, CKD contributes substantially to non-malignant causes of morbidity and mortality. Although the estimated glomerular filtration rate (eGFR) is widely used as a clinical indicator of renal function, robust imaging biomarkers for non-invasive assessment of renal dysfunction and disease severity remain poorly defined. Thus, there is a strong need for non-invasive biomarkers capable of assessing renal dysfunction before overt functional decline becomes apparent.

Current clinical monitoring of CKD relies on serial measurements of serum creatinine and eGFR, with sustained declines over two to three years, which are often employed as surrogate markers for progression to ESRD [5,6]. However, these approaches require long-term observations and cannot reliably forecast outcomes before a measurable loss of function occurs. Renal biopsy provides detailed histopathological and molecular information and remains the gold standard for the diagnosis of many kidney diseases; however, its invasiveness limits its repeated use and broad applicability [7].

Ischemia and fibrosis are the central pathophysiological drivers of progressive CKD [8,9]. Advanced MRI techniques hold promise as non-invasive tools for visualizing and quantifying tissue-level changes without a biopsy [10-12]. However, the relationship between MRI-derived metrics and the severity of renal dysfunction is not completely understood, and the translation of MRI biomarkers into clinical practice requires further investigation.

Diffusion-weighted MRI (DWI) traditionally uses the apparent diffusion coefficient (ADC) to reflect combined diffusion and microperfusion effects [13]. However, in tissues with substantial microvascular perfusion, such as the kidney, a biexponential intravoxel incoherent motion (IVIM) model may provide superior characterization [14]. IVIM acquires diffusion-weighted images at multiple low b-values to separate the true molecular diffusion (D), pseudo-perfusion diffusion coefficient (D*) related to microcapillary perfusion, and perfusion fraction (f). These parameters permit the distinct assessment of diffusion and perfusion components, and have gained attention in oncologic, neurologic, and musculoskeletal imaging as quantitative biomarkers and potential indicators of treatment response. In renal research, only preliminary small-scale IVIM studies have been reported [14], and comprehensive evaluations correlating IVIM metrics with eGFR and the severity of renal dysfunction are lacking.

Texture analysis (TA) quantifies intra-regional image heterogeneity by extracting mathematical features that describe pixel intensity distribution, spatial relationships, and structural patterns [15,16]. When combined with machine learning (ML), quantitative texture features (TFs) can be used to classify disease states and support quantitative disease assessments. Supervised learning algorithms (e.g., support vector machines and random forests) can select informative features, model complex non-linear relationships, and produce classifiers or prognostic scores. TA has been successfully applied in various medical imaging fields [17,18], demonstrating its versatility in detecting subtle tissue changes that are invisible to the human eye. In the renal context, TA may reveal the microarchitectural heterogeneity associated with ischemia, fibrosis, or inflammatory changes that precede a measurable decline in eGFR [19,20]. Therefore, integrating texture-derived descriptors with IVIM perfusion and diffusion metrics could enhance the model performance for the MRI-based assessment of CKD-related structural heterogeneity.

In this study, we aimed to investigate whether TA applied to IVIM-derived parameters provides incremental value over conventional ADC for the MRI-based assessment of renal dysfunction severity. We hypothesized that combining these complementary imaging biomarkers would improve the discrimination of renal dysfunction severity across CKD stages and provide a non-invasive imaging-based assessment tool in a clinical setting.

## Materials and methods

Participants

This study was approved by the Research Ethics Committee of Saitama Medical University Hospital (Saitama, Japan; approval number 2023-100). All experiments were conducted in accordance with relevant guidelines and regulations. The requirement for informed consent was waived due to the retrospective nature of the study.

Patients referred from the Department of Nephrology of our hospital who underwent kidney MRI between January 2008 and December 2011 were identified and reviewed. Inclusion criteria were (1) age of ≥15 years and (2) patients who underwent MRI scans, including IVIM sequences. The exclusion criteria were (1) incomplete execution of essential MRI scans; (2) insufficient clinical or imaging data; (3) advanced renal atrophy, making segmentation unfeasible; (4) severe artifacts on MRI; (5) presence of renal lesions with a maximum diameter exceeding 1 cm or more than five renal masses per kidney, including those associated with polycystic kidney disease; and (6) intervals of more than four weeks between the evaluation of eGFR and MRI scans.

A total of 79 participants were enrolled in the study, of whom 11 were excluded because of prominent imaging artifacts. 

The eGFR was calculated using the following equations:

$eGFR (mL/min/1.73 m^{2}) =194 &times; sCr^{&minus;1.094} &times; age^{&minus;0.287}$ (for men).

$eGFR (mL/min/1.73 m^{2}) =194 &times; sCr^{&minus;1.094} &times; age^{&minus;0.287}&times;0.739$ (for women)

The patients were stratified into three groups based on eGFR: the severe renal dysfunction (se-RD) (eGFR < 30 mL/min/1.73 m²; corresponding to CKD stages G4-G5), moderate renal dysfunction (mo-RD) (eGFR between 30 and 59 mL/min/1.73 m²; corresponding to CKD stages G3a-G3b), and mild renal dysfunction (mi-RD) (eGFR ≥ 60 mL/min/1.73 m²; corresponding to CKD stages G1-G2) groups.

MRI acquisition

MRI was performed using a 1.5-Tesla MRI Machine (Magnetom Sonata; Siemens Healthcare, Forchheim, Germany). The imaging protocol included DWI with eight b-values (b=0, 50, 100, 150, 300, 500, 700, and 900 s/mm^2^). The specific implementation protocol was as follows: repetition time, 5900 ms; echo time, 74 ms; flip angle, 90°; slice thickness 5.0 mm; acquisition matrix, 256 × 256; pixel bandwidth, 1500 Hz/pixel; field of view 40.0 cm.

ADC maps were automatically generated using the monoexponential model shown below, based on the DWI acquired at eight different b-values.

$Sb/S0=exp (-b&times;ADC)$

Similarly, the IVIM was acquired at eight different b values using the following equation:

$Sb/S0=Fp&times;exp(-b&times;D*)+(1-Fp)&times;exp(-b&times;D)$

Data analysis procedures

For each patient, two-dimensional segmentation was performed on a single corresponding coronal slice for each imaging method (ADC, D, D*, and f map), followed by feature selection and ML-based model development in separate classification attempts.

Segmentation

**Figure 1:**
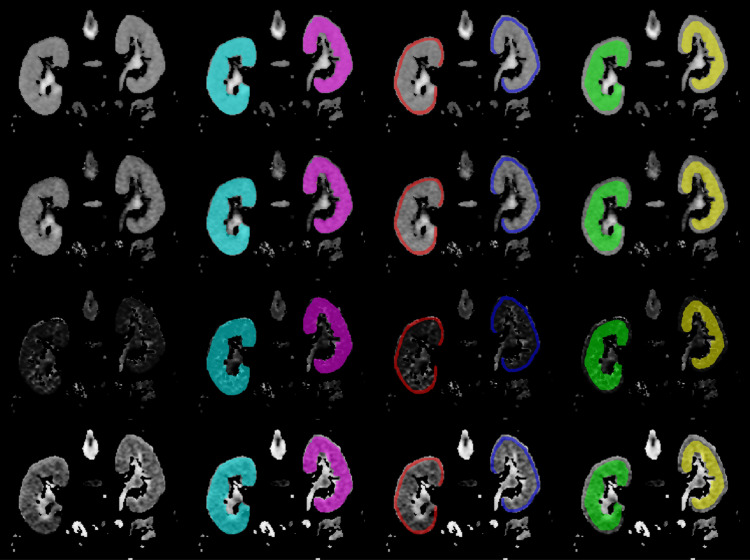
Representative images showing kidney region segmentation in apparent diffusion coefficient (ADC), true diffusion coefficient (D), pseudo-perfusion diffusion coefficient (D*), and perfusion fraction (f) maps. Each row, from top to bottom, represents ADC, D, D*, and f map images. Each column, from left to right, represents the original image, whole kidney segmentation (right kidney: light blue and left kidney: pink), renal cortex segmentation (right kidney: red and left kidney: blue), and renal medulla segmentation (right kidney: light green and left kidney: yellow).

After the MRI data were loaded onto an open-source software (ITK-SNAP version 3.8) [21], one slice of the images (ADC, D, D*, and f map) in the coronal plane was selected for each patient. For each patient, a single representative coronal slice was selected for analysis. Given the retrospective nature of the study and the variable image quality of the dataset, this approach was adopted to minimize motion-related artifacts and ensure consistency across imaging modalities. On each image, a manually drawn, irregular two-dimensional region of interest (ROI) was delineated to encompass the entire renal contour bilaterally while excluding cystic areas as much as possible (Figure 1).

Additionally, separate segmentations of the left and right kidneys as well as the renal cortex and medulla of both kidneys were performed to evaluate the classification performance of the TA models extracted from these regions. Segmentation of the ADC, D, D*, and f maps was performed independently by each radiologist with the clinical information for each case blinded. The region of interest (ROI) to be used for texture analysis was determined by consensus between the two radiologists.

TF Extraction

First, all MRI datasets underwent intensity normalization (scaling image intensity values to a range of 0-100) and were resampled to a uniform voxel size of 3 mm × 3 mm × 3 mm before feature extraction to mitigate data heterogeneity. Intensity normalization was performed with the following settings: normalizeScale = 100; voxelArrayShift = 300; force2D = True; ResampledPixelSpacing = (3, 3, 3); sigma = (3.0, 5.0); binWidth = 5.

We then computed TFs using an open-source software package capable of extracting a large panel of features from medical images (PyRadiomics version 2.1.0) [22]. The TFs were calculated based on six feature classes (first-order statistics, gray-level co-occurrence matrix (GLCM), gray level dependence matrix (GLDM), gray-level run-length matrix (GLRLM), gray level size zone matrix (GLSZM), and neighboring gray-tone difference matrix (NGTDM)).

First-order statistics were computed using the pixel-value histogram of the image. The first-order statistical features were energy, kurtosis, 10th percentile, 90th percentile, entropy, robust mean absolute deviation, interquartile range, maximum, mean, mean absolute deviation, median, minimum, range, root mean square, skewness, total energy, variance, and uniformity.

The GLCM calculates the occurrence of pairs of pixels with a specific value in a specific direction. In this study, the GLCM was calculated using a two-dimensional symmetric approach. The distance was set to one, considering pixels at a distance of 1 pixel from each other. The direction was divided into four: 0°, 45°, 90°, and 135°. This method can extract 24 statistical features: autocorrelation, cluster tendency, cluster shade, cluster prominence, contrast, correlation, difference variance, difference average, difference entropy, informational measure of correlation-1, informational measure of correlation-2, inverse variance, inverse difference normalized, inverse difference moment normalized, inverse difference moment, inverse difference moment, inverse difference moment, joint entropy, joint entropy, joint energy, maximal correlation coefficient, maximum probability, sum average, sum of squares, and sum entropy.

GLDM is a method for measuring the number of connected pixels within the distance of a center pixel. The distance between the center pixel and connected pixel was set to one. The cutoff value for dependence is by default set to zero. This method can extract 14 statistical features: small dependence emphasis, large dependence emphasis, small dependence low gray level emphasis, gray level non-uniformity, dependence non-uniformity, low gray level emphasis, high gray level emphasis, dependence entropy, dependence non-uniformity normalized, gray level variance, dependence variance, large dependence high gray level emphasis, large dependence low gray level emphasis, and small dependence high gray level emphasis.

The GLRLM is a method for defining the length/number of consecutive pixels (runs) that have the same gray-level value along an angle in the image. By default, the value of a feature was calculated separately in the GLRLM for each angle, after which the mean of these values was returned. This matrix can extract 16 statistical features: gray level non-uniformity, gray level non-uniformity normalized, low gray level run emphasis, short run emphasis, long run low gray level emphasis, long run high gray level emphasis, gray level variance, high gray level run emphasis, long run emphasis, run entropy, run length non-uniformity, run length non-uniformity normalized, run percentage, run variance, short run low gray level emphasis, and short run high gray level emphasis.

The GLSZM is used to quantify several pixels whose values have the same gray level (zone). This method can extract 16 features: gray level nonuniformity, gray level nonuniformity normalized, gray level variance, high gray level zone emphasis, large area emphasis, large area high gray level emphasis, large area low gray level emphasis, low gray level zone emphasis, size zone nonuniformity, size zone nonuniformity normalized, small area emphasis, small area high gray level emphasis, small area low gray level emphasis, zone entropy, zone percentage, and zone variance.

The NGTDM quantifies the difference between the gray value and the average gray value of its neighbors within a distance. The distance was set to one by default. This method extracts five statistical features: business, coarseness, complexity, contrast, and strength. A total of 93 TFs were extracted and analyzed to select the most valuable features for discriminating CKD grades using each imaging method. Textural features were computed on a slice-by-slice basis and then averaged. For the details of the 93 TFs described above, we referred to and described those listed in the official PyRadiomics documentation [22].

Dimensionality Reduction and Feature Selection

To mitigate the risk of overfitting and enhance the generalizability of the classification models, a dimensionality reduction of the TFs was performed. All the feature values were standardized using z-score normalization. Inter-observer reproducibility was assessed by calculating the intraclass correlation coefficient (ICC), and features demonstrating low reproducibility (ICC <0.75 or lower bound of the 95% confidence interval <0.6) in any imaging modality were excluded from further analysis.

Subsequently, feature selection was performed using the sequential feature selection (SFS) algorithm, which is a wrapper-based greedy search method that iteratively adds or removes features to optimize model performance based on a predefined classifier. Six representative ML classifiers were employed: linear discriminant analysis (LDA), quadratic discriminant analysis (QDA), support vector machine (SVM), k-nearest neighbors (k-NN), decision tree (DT), and random forest (RF). The rbf kernel was used as the SVM classifier.

Using the SFS algorithm, the optimal subset of features for each classifier was identified, and the number of selected TFs was limited to three to prevent overfitting due to the relatively small sample size.

Classification and Validation

Multiclass classification models were constructed using the six ML classifiers: LDA, QDA, SVM, k-NN, DT, and RF. We validated the performance of these models using a cross-validation method. The 10-fold cross-validation method was repeated 100 times to retain the stability and reproducibility of the results. The performance of the classifiers was evaluated using receiver operating characteristic (ROC) analysis and the area under the curve (AUC) as the main performance metric. The accuracy, precision, recall, and f-measure for each group and the macro average for all groups were calculated. For the multiclass AUC, we employed a one-vs-rest approach, and AUCs were computed using a macro average across classes. We also evaluated the performance of the classification models for TFs extracted from the left and right kidneys, as well as the renal cortex and medulla of both kidneys, and compared the differences between cortex and medulla as the secondary metric. Statistical analyses were performed using an open-source software package (Python scikit-learn 0.22.1) [23].

## Results

Clinical and demographic data

The study included 68 participants. The major underlying etiologies of CKD were as follows: hypertensive nephrosclerosis in 28 patients, diabetic nephropathy in 11, and chronic glomerulonephritis in 17 (including five with IgA nephropathy, five with nephrotic syndrome, one with membranous nephropathy, and six with other subtypes). Additionally, interstitial nephritis was observed in two patients, anti-neutrophil cytoplasmic antibody-associated (ANCA)-associated vasculitis in one, lupus nephritis in one, and nodular glomerulosclerosis in one; the etiology remained unidentified in 14 patients.

According to the eGFR, 23 patients had se-RD (male participants, n (%) = 9 (39), mean age 59.0 ± 16.6 years, mean eGFR 14.0 ± 11.9 mL/min/1.73 m^2^), 19 patients had mo-RD (male participants, n (%) = 11 (58), mean age 56.8 ± 13.7 years, mean eGFR 47.0 ± 13.9 mL/min/1.73 m^2^), and 26 patients had mi-RD (male participants, n (%) = 14 (54), mean age 36.5 ± 17.3 years, mean eGFR 82.3 ± 30.0 mL/min/1.73 m^2^).

Regarding etiology, among 23 patients with severe renal impairment, hypertensive nephrosclerosis was observed in 19 patients and diabetic nephropathy in five. Chronic glomerulonephritis was diagnosed in two patients; of these, one had IgA nephropathy and one had nephrotic syndrome. The etiology was unknown in two patients.

Among 19 patients with moderate renal impairment, hypertensive nephrosclerosis was present in seven patients, diabetic nephropathy in five, interstitial nephritis in one, and ANCA‑associated vasculitis in one. Chronic glomerulonephritis was diagnosed in six patients; three of these presented with nephrotic syndrome. The etiology was unknown in three patients.

Among 26 patients with mild renal impairment, hypertensive nephrosclerosis was identified in two patients, diabetic nephropathy in one, interstitial nephritis in one, and lupus nephritis in one. Chronic glomerulonephritis was diagnosed in nine patients; of these, four had IgA nephropathy, one had nephrotic syndrome, and one had membranous nephropathy. The etiology was unknown in nine patients.

Dimension reduction of TFs

Most TFs showed acceptable reproducibility in the inter-observer reproducibility analysis. The ICC values for all TFs are summarized in Table 1. 

**Table 1:** The inter-observer reproducibility test for each subset of texture features The intraclass correlation coefficient was calculated to evaluate the inter-observer reproducibility for each subset of texture features. The feature classes were as follows: first-order statistics, gray-level co-occurrence matrix (GLCM), gray-level dependence matrix (GLDM), gray-level run-length matrix (GLRLM), gray-level size zone matrix (GLSZM), and neighboring gray-tone difference matrix (NGTDM). The imaging sequences were as follows: apparent diffusion coefficient (ADC), true diffusion coefficient (D), pseudo-perfusion diffusion coefficient (D*), and perfusion fraction (f).

	ADC	D	D*	f
10th percentile (first-order)	0.826	0.899	0.966	0.833
90th percentile (first-order)	0.996	0.999	0.969	0.971
Energy (first-order)	0.962	0.978	0.806	0.656
Entropy (first-order)	0.724	0.707	0.700	0.664
Interquartile range (first-order)	0.701	0.707	0.701	0.661
Kurtosis (first-order)	0.922	0.706	0.864	0.749
Maximum (first-order)	0.995	0.985	0.870	0.873
Mean absolute deviation (first-order)	0.714	0.706	0.701	0.652
Mean (first-order)	0.991	0.997	0.963	0.946
Median (first-order)	0.719	0.703	0.763	0.683
Minimum (first-order)	0.938	0.865	0.847	0.694
Range (first-order)	0.996	0.992	0.945	0.762
Robust mean absolute deviation (first-order)	0.734	0.708	0.701	0.749
Root mean squared (first-order)	0.846	0.885	0.853	0.849
Skewness (first-order)	0.720	0.717	0.932	0.694
Total energy (first-order)	0.962	0.978	0.951	0.656
Uniformity (first-order)	0.754	0.703	0.700	0.651
Variance (first-order)	0.997	0.997	0.697	0.693
Autocorrelation (GLCM)	0.996	0.999	0.852	0.840
Cluster prominence (GLCM)	0.999	0.999	0.945	0.648
Cluster shade (GLCM)	0.998	0.999	0.951	0.653
Cluster tendency (GLCM)	0.988	0.999	0.865	0.665
Contrast (GLCM)	0.999	0.998	0.859	0.785
Correlation (GLCM)	0.741	0.721	0.700	0.716
Difference average (GLCM)	0.987	0.986	0.657	0.646
Difference entropy (GLCM)	0.762	0.707	0.681	0.655
Difference variance (GLCM)	0.999	0.999	0.808	0.751
Inverse difference (GLCM)	0.734	0.701	0.756	0.666
Inverse difference moment (GLCM)	0.838	0.700	0.671	0.652
Inverse difference moment normalized (GLCM)	0.713	0.713	0.652	0.724
Inverse difference normalized (GLCM)	0.744	0.701	0.660	0.711
Informational measure of correlation-1 (GLCM)	0.738	0.700	0.700	0.731
Informational measure of correlation-2 (GLCM)	0.797	0.700	0.661	0.736
Inverse variance (GLCM)	0.833	0.863	0.894	0.882
Joint average (GLCM)	0.987	0.990	0.918	0.775
Joint energy (GLCM)	0.772	0.700	0.901	0.701
Joint entropy (GLCM)	0.776	0.999	0.955	0.685
Maximal correlation coefficient (GLCM)	0.754	0.859	0.867	0.824
Maximum probability (GLCM)	0.994	0.891	0.918	0.806
Sum average (GLCM)	0.980	0.986	0.877	0.774
Sum entropy (GLCM)	0.929	0.924	0.656	0.763
Sum squares (GLCM)	0.997	0.997	0.670	0.857
Dependence entropy (GLDM)	0.897	0.828	0.718	0.885
Dependence non uniformity (GLDM)	0.882	0.890	0.690	0.866
Dependence non uniformity normalized (GLDM)	0.720	0.702	0.680	0.704
Dependence variance (GLDM)	0.985	0.995	0.799	0.682
Gray level non uniformity (GLDM)	0.999	0.979	0.712	0.732
Gray level variance (GLDM)	0.997	0.997	0.696	0.792
High gray level emphasis (GLDM)	0.995	0.998	0.851	0.841
Large dependence emphasis (GLDM)	0.966	0.860	0.738	0.707
Large dependence high gray level emphasis (GLDM)	0.997	0.999	0.653	0.680
Large dependence low gray level emphasis (GLDM)	0.858	0.702	0.701	0.653
Low gray level emphasis (GLDM)	0.713	0.703	0.702	0.661
Small dependence emphasis (GLDM)	0.797	0.700	0.762	0.722
Small dependence high gray level emphasis (GLDM)	0.995	0.999	0.898	0.916
Small dependence low gray level emphasis (GLDM)	0.725	0.700	0.651	0.707
Gray level non uniformity (GLRLM)	0.988	0.998	0.786	0.825
Gray level non uniformity normalized (GLRLM)	0.702	0.704	0.705	0.706
Gray level variance (GLRLM)	0.998	0.973	0.710	0.828
High gray level run emphasis (GLRLM)	0.997	0.998	0.849	0.843
Long run emphasis (GLRLM)	0.702	0.700	0.670	0.675
Long run high gray level emphasis (GLRLM)	0.997	0.998	0.769	0.673
Long run low gray level emphasis (GLRLM)	0.999	0.999	0.748	0.846
Low gray level run emphasis (GLRLM)	0.921	0.900	0.709	0.710
Run entropy (GLRLM)	0.715	0.700	0.643	0.701
Run length non uniformity (GLRLM)	0.907	0.846	0.711	0.727
Run length non uniformity normalized (GLRLM)	0.701	0.700	0.722	0.741
Run percentage (GLRLM)	0.912	0.950	0.726	0.839
Run variance (GLRLM)	0.732	0.700	0.644	0.701
Short run emphasis (GLRLM)	0.702	0.700	0.701	0.670
Short run high gray level emphasis (GLRLM)	0.997	0.998	0.874	0.861
Short run low gray level emphasis (GLRLM)	0.703	0.701	0.699	0.712
Gray level non uniformity (GLSZM)	0.919	0.863	0.655	0.733
Gray level non uniformity normalized (GLSZM)	0.991	0.990	0.670	0.747
Gray level variance (GLSZM)	0.999	0.997	0.866	0.837
High gray level zone emphasis (GLSZM)	0.983	0.778	0.841	0.826
Large area emphasis (GLSZM)	0.999	0.999	0.738	0.892
Large area high gray level emphasis (GLSZM)	0.998	0.998	0.812	0.721
Large area low gray level emphasis (GLSZM)	0.999	0.999	0.733	0.876
Low gray level zone emphasis (GLSZM)	0.701	0.801	0.651	0.704
Size zone non uniformity (GLSZM)	0.951	0.923	0.648	0.761
Size zone non uniformity normalized (GLSZM)	0.827	0.938	0.809	0.694
Small area emphasis (GLSZM)	0.703	0.700	0.703	0.650
Small area high gray level emphasis (GLSZM)	0.999	0.998	0.878	0.885
Small area low gray level emphasis (GLSZM)	0.702	0.700	0.687	0.660
Zone entropy (GLSZM)	0.701	0.702	0.703	0.633
Zone percentage (GLSZM)	0.732	0.705	0.700	0.651
Zone variance (GLSZM)	0.999	0.701	0.691	0.651
Busyness (NGTDM)	0.986	0.927	0.689	0.641
Coarseness (NGTDM)	0.751	0.830	0.650	0.650
Complexity (NGTDM)	0.999	0.999	0.822	0.641
Contrast (NGTDM)	0.999	0.853	0.653	0.714
Strength (NGTDM)	0.999	0.999	0.680	0.708

Among the 93 TFs, the number of those with an ICC ≥0.8 was 59, 58, 34, and 25 for the ADC, D, D*, and f maps, respectively. In contrast, the numbers of features with ICC ≥0.6 and <0.8 were 34, 35, 59, and 68 for the ADC, D, D*, and f maps, respectively.

Subsequently, a subset of the three TFs was selected for each renal compartment and imaging sequence using the SFS algorithm. The features selected for each classification are listed in Table 2.

**Table 2:** Selected texture features derived from bilateral kidneys for classifying the grades of chronic kidney disease The feature classes were as follows: first-order statistics, gray-level co-occurrence matrix (GLCM), gray-level dependence matrix (GLDM), gray-level run-length matrix (GLRLM), gray-level size zone matrix (GLSZM), and neighboring gray-tone difference matrix (NGTDM). The imaging sequences were as follows: apparent diffusion coefficient (ADC), true diffusion coefficient (D), pseudo-perfusion diffusion coefficient (D*), and perfusion fraction (f). DT, decision tree; k-NN, k-nearest neighbors; LDA, linear discriminant analysis; QDA, quadratic discriminant analysis; RF, random forest; SVM, support vector machine.

	LDA	QDA	SVM	k-NN	DT	RF
ADC	Energy (first-order)	Informational measure of correlation-1 (GLCM)	Large dependence high gray level emphasis (GLDM)	Inverse difference moment normalized (GLCM)	Maximum (first-order)	Total energy (first-order)
	Inverse difference normalized (GLCM)	Large dependence high gray level emphasis (GLDM)	Low gray level emphasis (GLDM)	Inverse difference normalized (GLCM)	Total energy (first-order)	Large dependence emphasis (GLDM)
	Gray level non uniformity (GLDM)	Gray level non uniformity (GLRLM)	Low gray level zone emphasis (GLSZM)	Large dependence emphasis (GLDM)	Large area high gray level emphasis (GLSZM)	Large area high gray level emphasis (GLSZM)
D	Energy (first-order)	Maximal correlation coefficient (GLCM)	Minimum (first-order)	Minimum (first-order)	Inverse difference moment normalized (GLCM)	Range (first-order)
	Cluster prominence (GLCM)	Run length non uniformity (GLRLM)	Cluster prominence (GLCM)	Dependence non uniformity (GLDM)	Dependence non uniformity (GLDM)	Dependence non uniformity (GLDM)
	Large area emphasis (GLSZM)	Low gray level zone emphasis (GLSZM)	Dependence non uniformity (GLDM)	Small area low gray level emphasis (GLSZM)	Large dependence emphasis (GLDM)	Low gray level zone emphasis (GLSZM)
D*	Energy (first-order)	Median (first-order)	Variance (first-order)	Run length non uniformity (GLRLM)	Variance (first-order)	Energy (first-order)
	Dependence non uniformity (GLDM)	Run length non uniformity normalized (GLRLM)	High gray level emphasis (GLDM)	Large area high gray level emphasis (GLSZM)	High gray level zone emphasis (GLSZM)	Cluster shade (GLCM)
	Dependence non uniformity normalized (GLDM)	Complexity (NGTDM)	Gray level non uniformity (GLSZM)	Zone entropy (GLSZM)	Size zone non uniformity (GLSZM)	Dependence non uniformity normalized (GLDM)
f	Skewness (first-order)	Correlation (GLCM)	Inverse variance (GLCM)	Entropy (first-order)	Cluster shade (GLCM)	Dependence entropy (GLDM)
	Informational Measure of Correlation-2 (GLCM)	Run length non uniformity (GLRLM)	Dependence non uniformity (GLDM)	Dependence non uniformity (GLDM)	Correlation (GLCM)	Dependence non uniformity (GLDM)
	Run length non uniformity (GLRLM)	High gray level zone emphasis (GLSZM)	Run variance (GLRLM)	Gray level non uniformity (GLSZM)	Dependence entropy (GLDM)	Low gray level emphasis (GLDM)

Classification model using the TFs from bilateral kidneys

Overall, our MRI-based TA models provided acceptable to good discriminatory performance across the classification attempts, as shown in Table 3.

**Table 3:** Results for the classification model using the texture features from bilateral kidneys Imaging sequences were as follows: apparent diffusion coefficient (ADC), true diffusion coefficient (D), pseudo-perfusion diffusion coefficient (D*), and perfusion fraction (f). DT, decision tree; k-NN, k-nearest neighbors; LDA, linear discriminant analysis; QDA, quadratic discriminant analysis; RF, random forest; SVM, support vector machine.

		Accuracy	Precision	Recall	F-measure	ROC AUC
ADC	LDA	0.681 ± 0.021	0.675 ± 0.027	0.661 ± 0.023	0.658 ± 0.026	0.781 ± 0.009
	QDA	0.709 ± 0.019	0.692 ± 0.021	0.690 ± 0.020	0.686 ± 0.021	0.851 ± 0.010
	SVM	0.618 ± 0.027	0.611 ± 0.056	0.583 ± 0.028	0.554 ± 0.036	0.705 ± 0.020
	k-NN	0.370 ± 0.029	0.364 ± 0.031	0.371 ± 0.029	0.361 ± 0.030	0.562 ± 0.023
	DT	0.622 ± 0.039	0.617 ± 0.039	0.613 ± 0.039	0.613 ± 0.039	0.712 ± 0.024
	RF	0.669 ± 0.025	0.637 ± 0.039	0.638 ± 0.026	0.616 ± 0.031	0.788 ± 0.012
D	LDA	0.614 ± 0.023	0.567 ± 0.038	0.580 ± 0.025	0.555 ± 0.029	0.733 ± 0.015
	QDA	0.550 ± 0.027	0.525 ± 0.038	0.525 ± 0.028	0.524 ± 0.032	0.676 ± 0.018
	SVM	0.566 ± 0.023	0.412 ± 0.045	0.522 ± 0.023	0.455 ± 0.022	0.642 ± 0.022
	k-NN	0.557 ± 0.023	0.529 ± 0.026	0.533 ± 0.024	0.526 ± 0.025	0.681 ± 0.016
	DT	0.519 ± 0.039	0.516 ± 0.040	0.511 ± 0.039	0.510 ± 0.039	0.632 ± 0.030
	RF	0.552 ± 0.019	0.515 ± 0.030	0.523 ± 0.020	0.506 ± 0.023	0.718 ± 0.019
D*	LDA	0.683 ± 0.021	0.676 ± 0.026	0.663 ± 0.022	0.661 ± 0.024	0.780 ± 0.010
	QDA	0.459 ± 0.022	0.439 ± 0.031	0.436 ± 0.021	0.414 ± 0.022	0.614 ± 0.011
	SVM	0.450 ± 0.019	0.349 ± 0.055	0.417 ± 0.018	0.366 ± 0.022	0.588 ± 0.026
	k-NN	0.521 ± 0.023	0.490 ± 0.027	0.501 ± 0.024	0.492 ± 0.025	0.641 ± 0.016
	DT	0.487 ± 0.039	0.487 ± 0.040	0.487 ± 0.041	0.485 ± 0.040	0.611 ± 0.032
	RF	0.527 ± 0.032	0.508 ± 0.044	0.505 ± 0.034	0.497 ± 0.038	0.621 ± 0.018
f	LDA	0.636 ± 0.022	0.618 ± 0.026	0.614 ± 0.023	0.605 ± 0.025	0.755 ± 0.008
	QDA	0.593 ± 0.028	0.580 ± 0.031	0.579 ± 0.029	0.577 ± 0.030	0.722 ± 0.015
	SVM	0.555 ± 0.025	0.526 ± 0.033	0.533 ± 0.028	0.524 ± 0.031	0.671 ± 0.022
	k-NN	0.556 ± 0.033	0.549 ± 0.030	0.550 ± 0.032	0.545 ± 0.032	0.748 ± 0.013
	DT	0.424 ± 0.043	0.424 ± 0.044	0.424 ± 0.042	0.422 ± 0.043	0.569 ± 0.031
	RF	0.637 ± 0.027	0.614 ± 0.037	0.613 ± 0.028	0.601 ± 0.031	0.752 ± 0.015

**Figure 2:**
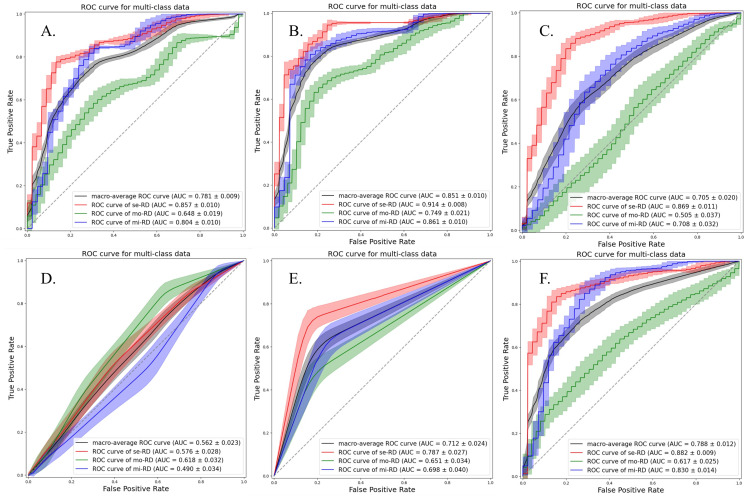
ROC curves and AUC values of each classification attempt for classifying the grades of chronic kidney disease severity using ADC map with texture features from bilateral kidneys Multiclass classification models were constructed using the following six machine learning classifiers: linear discriminant analysis (a), quadratic discriminant analysis (b), support vector machine (c), k-nearest neighbors (d), decision tree (e), and random forest (f). se-RD, severe renal disease; mi-RD, mild renal disease; mo-RD, moderate renal disease.

**Figure 3:**
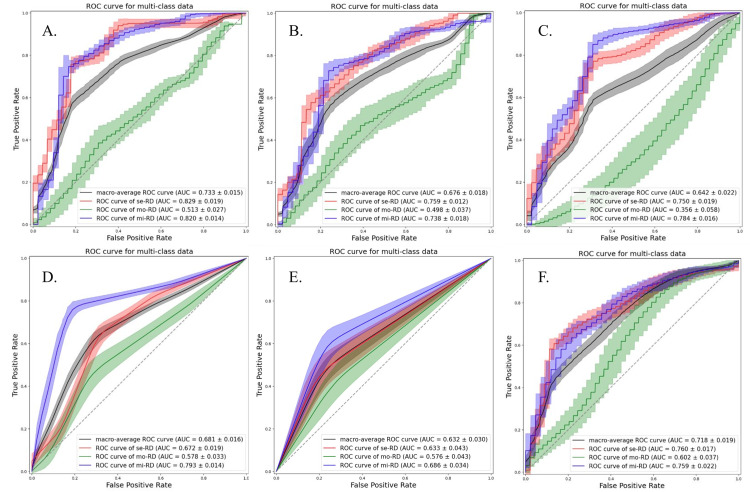
The ROC curves and AUC values of each classification attempt for classifying the grades of chronic kidney disease severity using true diffusion coefficient map with texture features from bilateral kidneys Multiclass classification models were constructed using the following six machine learning classifiers: linear discriminant analysis (a), quadratic discriminant analysis (b), support vector machine (c), k-nearest neighbors (d), decision tree (e), and random forest (f). se-RD, severe renal disease; mi-RD, mild renal disease; mo-RD, moderate renal disease; ROC, receiver receiver operating characteristic; AUC, area under the curve.

**Figure 4:**
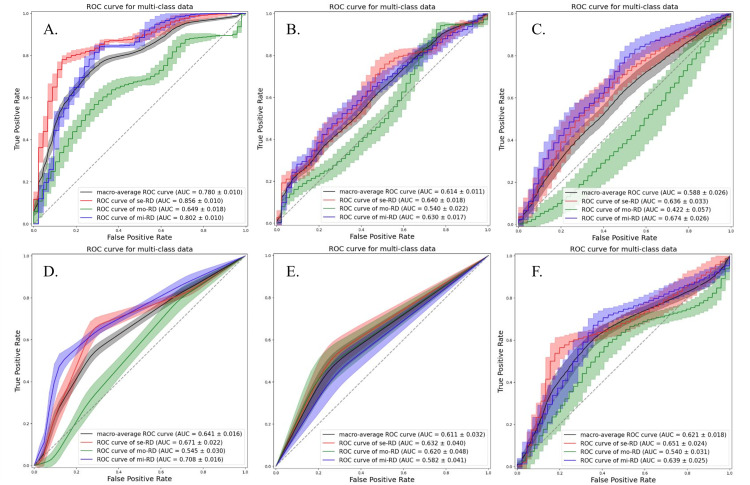
The ROC curves and AUC values of each classification attempt for classifying the grades of chronic kidney disease severity using pseudo-perfusion diffusion coefficient map with texture features from bilateral kidneys Multiclass classification models were constructed using the following six machine learning classifiers: linear discriminant analysis (a), quadratic discriminant analysis (b), support vector machine (c), k-nearest neighbors (d), decision tree (e), and random forest (f). se-RD, severe renal disease; mi-RD, mild renal disease; mo-RD, moderate renal disease; ROC, receiver operating characteristic; AUC, area under the curve; f, perfusion fraction.

**Figure 5:**
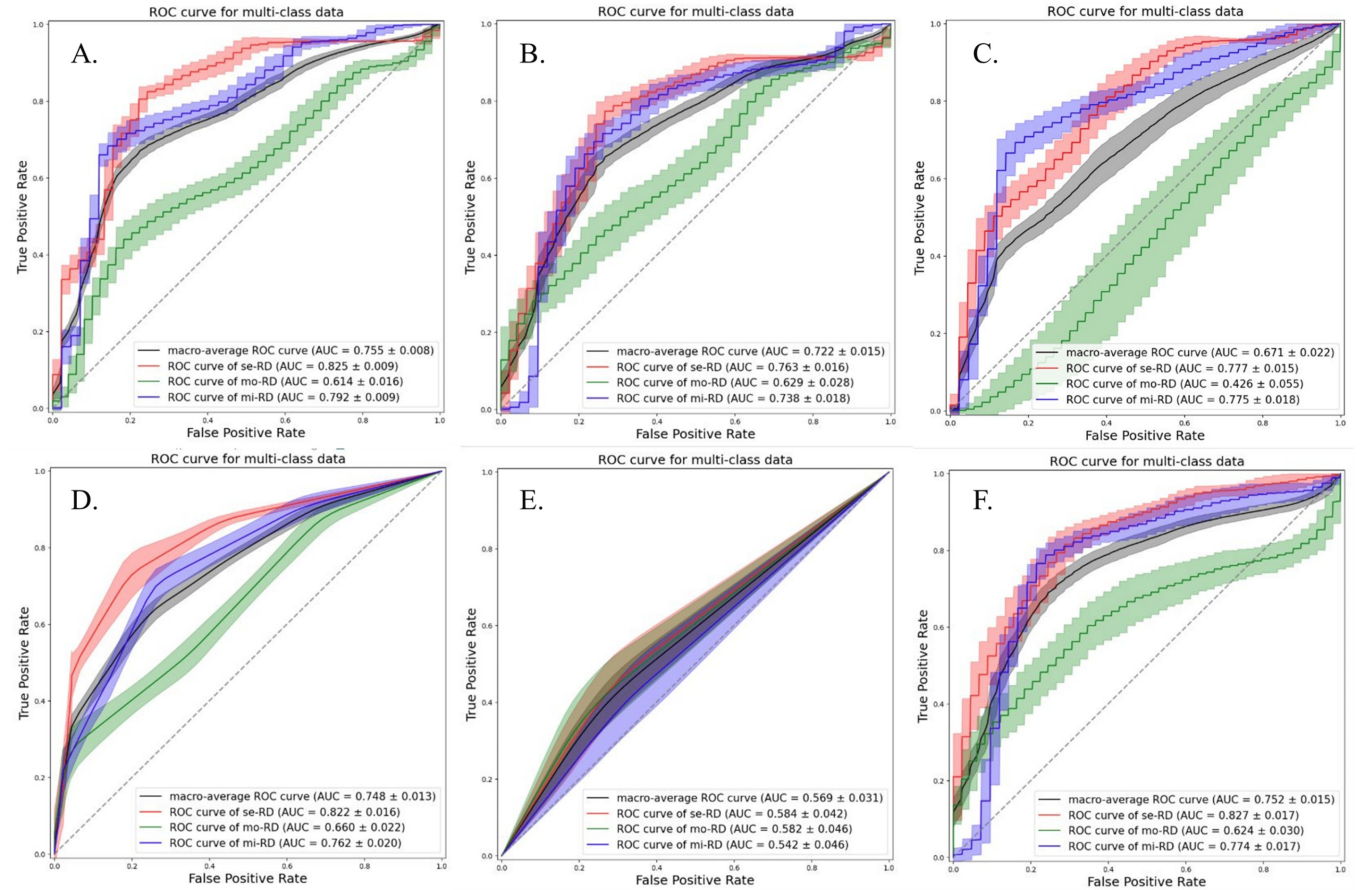
The ROC curves and AUC values of each classification attempt for classifying the grades of chronic kidney disease severity using perfusion fraction map with texture features from bilateral kidneys Multiclass classification models were constructed using the following six machine learning classifiers: linear discriminant analysis (a), quadratic discriminant analysis (b), support vector machine (c), k-nearest neighbors (d), decision tree (e), and random forest (f). se-RD, severe renal disease; mi-RD, mild renal disease; mo-RD, moderate renal disease; ROC, receiver operating characteristic; AUC, area under the curve.

Although the accuracies were almost similar for each ML classifier, the LDA classifier demonstrated a relatively stable performance. The models using the ADC map exhibited better performances than those derived from the D, D*, and f maps. The best performance was obtained when the ADC map and QDA classifier were used. Among the non-linear classifiers, the RF classifier exhibited a relatively stable performance. The ROC curves for all classification attempts are shown in Figures 2-5. 

**Figure 6:**
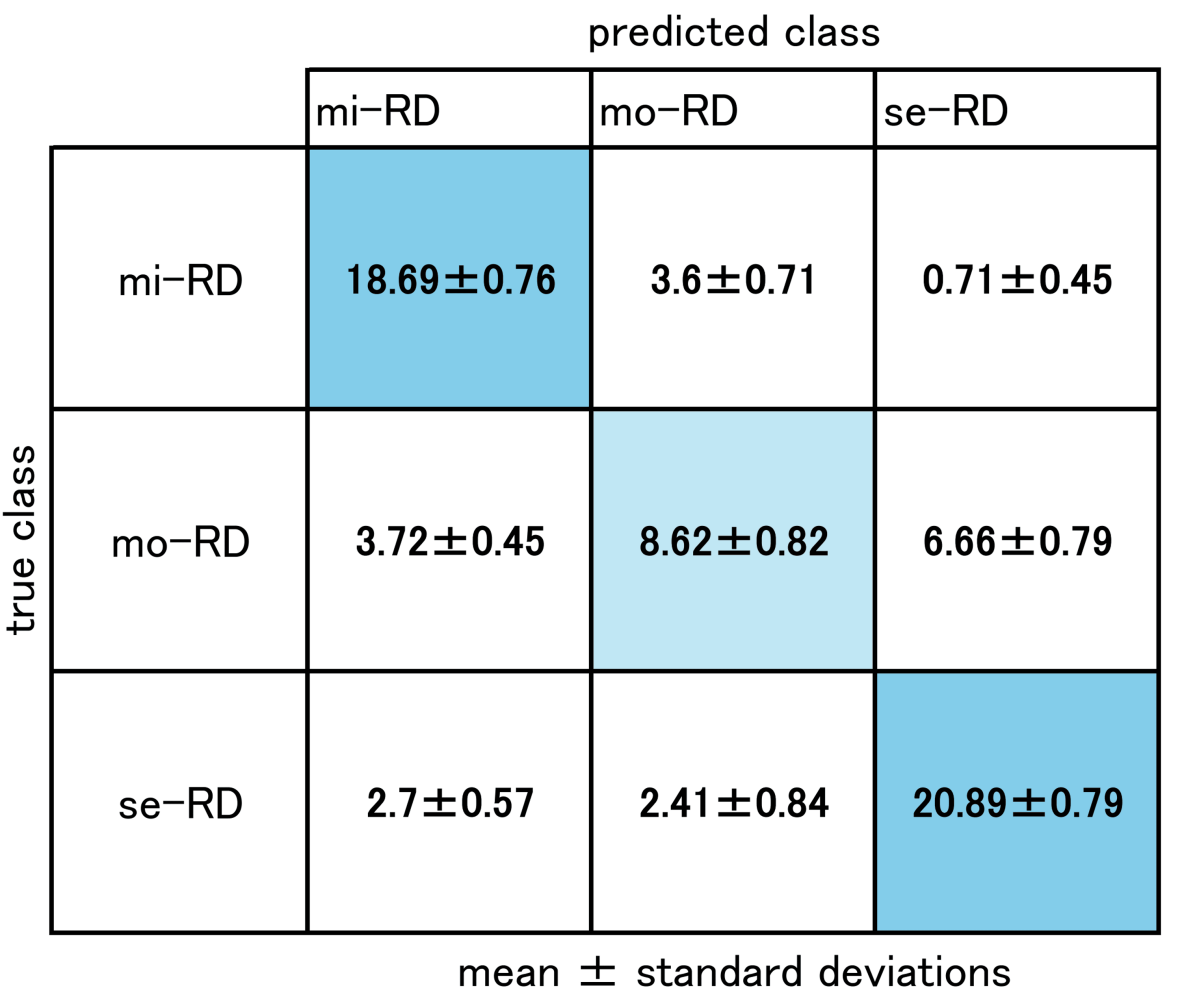
The confusion map of macro average and standard deviation of ADC map-based QDA model, which achieved the highest performance Each row, represents the predicted class, from top to bottom, mild renal disease (mi-RD), moderate renal disease (mo-RD), and severe renal disease (se-RD). Each column, represents the true class, left top to right, mi-RD, mo-RD, se-RD. The numbers in the table denote the mean and standard deviation of the macro-average of 100 times repeated 10-fold cross-validation, respectively. ADC, apparent diffusion coefficient; QDA, quadratic discriminant analysis.

Figure 6 shows the confusion matrix for the ADC map-based QDA model, which achieved the highest performance; values represent the macro‑average ± standard deviation.

Classification model using the TFs from the ipsilateral kidney and renal medulla/cortex

We developed classification models using representative classifiers (i.e., QDA and RF). The selected TFs and results of all classification attempts are summarized in Table 4 and Table 5, respectively.

**Table 4:** Selected texture features derived from the ipsilateral kidney and renal medulla/cortex for classifying the grades of chronic kidney disease The feature classes were as follows: first-order statistics, gray-level co-occurrence matrix (GLCM), gray-level dependence matrix (GLDM), gray-level run-length matrix (GLRLM), gray-level size zone matrix (GLSZM), and neighboring gray-tone difference matrix (NGTDM). The imaging sequences were as follows: apparent diffusion coefficient (ADC), true diffusion coefficient (D), pseudo-perfusion diffusion coefficient (D*), and perfusion fraction (f). QDA, quadratic discriminant analysis; RF, random forest.

		QDA	RF
Right whole kidney	ADC	Energy (first-order), Large area high gray level emphasis (GLSZM), Contrast (NGTDM)	Kurtosis (first-order), Dependence variance (GLDM), Small area emphasis (GLSZM)
	D	10th Percentile (first-order), Energy (first-order), Coarseness (NGTDM)	Maximum (first-order), Difference entropy (GLCM), Informational measure of correlation-2 (GLCM)
	D*	Maximum probability (GLCM), Dependence non uniformity (GLDM), Gray level non uniformity (GLRLM)	Root mean squared (first-order), Difference entropy (GLCM), Inverse difference normalized (GLCM)
	f	Minimum (first-order), Sum average (GLCM), Run length non uniformity (GLRLM)	Energy (first-order), Robust mean absolute deviation (first-order), Uniformity (first-order)
Left whole kidney	ADC	Energy (first-order), Small dependence high gray level emphasis (GLDM), Run length non uniformity (GLRLM)	Kurtosis (first-order), Run length non uniformity (GLRLM), Low gray level zone emphasis (GLSZM)
	D	10th Percentile (first-order), Energy (first-order), Coarseness (NGTDM)	Maximum (first-order), Difference entropy (GLCM), Gray level non uniformity (GLSZM)
	D*	10th Percentile (first-order), 90th Percentile (first-order), Robust mean absolute deviation (first-order)	Median (first-order), Zone variance (GLSZM), Contrast (NGTDM)
	f	Minimum (first-order), Sum average (GLCM), Run length non uniformity (GLRLM)	Energy (first-order), Uniformity (first-order), Run length non uniformity (GLRLM)
Right renal medulla	ADC	Energy (first-order), Large area emphasis (GLSZM), Large area high gray level emphasis (GLSZM)	Energy (first-order), Entropy (first-order), Maximum (first-order)
	D	90th Percentile (first-order), Energy (first-order), Large dependence high gray level emphasis (GLDM)	Mean (first-order), Difference entropy (GLCM), Inverse difference moment normalized (GLCM)
	D*	Minimum (first-order), Maximal correlation coefficient (GLCM), Gray level non uniformity (GLSZM)	Inverse difference normalized (GLCM), Joint average (GLCM), Gray level non uniformity (GLDM)
	f	Joint average (GLCM), Run length non uniformity (GLRLM), Coarseness (NGTDM)	Mean absolute deviation (first-order), Maximal correlation coefficient (GLCM), Dependence non uniformity (GLDM)
Left renal medulla	ADC	Energy (first-order), Run entropy (GLRLM), Size zone non uniformity (GLSZM)	Inverse difference moment (GLCM), Run length non uniformity (GLRLM), Coarseness (NGTDM)
	D	Energy (first-order), Inverse variance (GLCM), Run length non uniformity (GLRLM)	Mean absolute deviation (first-order), Difference entropy (GLCM), Dependence non uniformity (GLDM)
	D*	10th Percentile (first-order), 90th Percentile (first-order), Robust mean absolute deviation (first-order)	Difference entropy (GLCM), Dependence entropy (GLDM), Run length non uniformity (GLRLM)
	f	Cluster shade (GLCM), Difference average (GLCM), Size zone non uniformity (GLSZM)	Uniformity (first-order), Correlation (GLCM), Size zone non uniformity (GLSZM)
Right renal cortex	ADC	90th Percentile (first-order), Energy (first-order), Small area high gray level emphasis (GLSZM)	Median (first-order), Difference entropy (GLCM), Small area low gray level emphasis (GLSZM)
	D	Energy (first-order), Total energy (first-order), Dependence non uniformity (GLDM)	Difference entropy (GLCM), Large dependence emphasis (GLDM), Gray level non uniformity (GLSZM)
	D*	90th Percentile (first-order), Energy (first-order), Large dependence high gray level emphasis (GLDM)	Mean (first-order), Cluster shade (GLCM), Joint energy (GLCM)
	f	Energy (first-order), Dependence entropy (GLDM), Dependence non uniformity (GLDM)	Cluster prominence (GLCM), Inverse difference moment normalized (GLCM), Short run emphasis (GLRLM)
Left renal cortex	ADC	90th Percentile (first-order), Energy (first-order), Large dependence high gray level emphasis (GLDM)	Total energy (first-order), Correlation (GLCM), Small area emphasis (GLSZM)
	D	10th Percentile (first-order), Run length non uniformity (GLRLM), Large area high gray level emphasis (GLSZM)	Difference entropy (GLCM), Run length non uniformity (GLRLM), Small area low gray level emphasis (GLSZM)
	D*	Contrast (GLCM), Difference average (GLCM), Gray level non uniformity (GLSZM)	Correlation (GLCM), Gray level non uniformity normalized (GLRLM), Short run emphasis (GLRLM)
	f	Energy (first-order), Joint entropy (GLCM), Busyness (NGTDM)	Difference entropy (GLCM), Inverse difference moment normalized (GLCM), Small area low gray level emphasis (GLSZM)

**Table 5:** Results for the classification model using the texture features from the ipsilateral kidney and renal medulla/cortex Imaging sequences were as follows: apparent diffusion coefficient (ADC), true diffusion coefficient (D), pseudo-perfusion diffusion coefficient (D*), and perfusion fraction (f). QDA, quadratic discriminant analysis; RF, random forest; ROC, receiver operating characteristic; AUC, area under the curve.

			Accuracy	Precision	Recall	F-measure	ROC AUC
Right whole kidney	ADC	QDA	0.576 ± 0.025	0.524 ± 0.044	0.546 ± 0.025	0.512 ± 0.029	0.701 ± 0.018
		RF	0.574 ± 0.029	0.584 ± 0.049	0.555 ± 0.030	0.545 ± 0.035	0.744 ± 0.018
	D	QDA	0.532 ± 0.021	0.447 ± 0.034	0.495 ± 0.020	0.456 ± 0.022	0.626 ± 0.016
		RF	0.499 ± 0.026	0.425 ± 0.061	0.467 ± 0.026	0.422 ± 0.031	0.598 ± 0.020
	D*	QDA	0.549 ± 0.020	0.559 ± 0.021	0.552 ± 0.022	0.547 ± 0.020	0.684 ± 0.011
		RF	0.432 ± 0.037	0.346 ± 0.045	0.400 ± 0.036	0.364 ± 0.037	0.596 ± 0.023
	f	QDA	0.539 ± 0.029	0.522 ± 0.040	0.514 ± 0.030	0.504 ± 0.032	0.673 ± 0.016
		RF	0.536 ± 0.027	0.509 ± 0.059	0.506 ± 0.030	0.482 ± 0.040	0.668 ± 0.017
Left whole kidney	ADC	QDA	0.649 ± 0.023	0.635 ± 0.025	0.635 ± 0.022	0.629 ± 0.023	0.767 ± 0.013
		RF	0.590 ± 0.033	0.567 ± 0.041	0.570 ± 0.034	0.561 ± 0.037	0.733 ± 0.014
	D	QDA	0.561 ± 0.021	0.498 ± 0.035	0.527 ± 0.021	0.495 ± 0.025	0.705 ± 0.017
		RF	0.530 ± 0.021	0.394 ± 0.036	0.492 ± 0.020	0.432 ± 0.020	0.615 ± 0.018
	D*	QDA	0.436 ± 0.023	0.301 ± 0.019	0.398 ± 0.021	0.338 ± 0.017	0.468 ± 0.023
		RF	0.478 ± 0.030	0.406 ± 0.045	0.445 ± 0.031	0.415 ± 0.033	0.613 ± 0.019
	f	QDA	0.537 ± 0.031	0.522 ± 0.039	0.513 ± 0.031	0.503 ± 0.032	0.674 ± 0.013
		RF	0.559 ± 0.025	0.529 ± 0.036	0.533 ± 0.026	0.516 ± 0.029	0.688 ± 0.015
Right renal medulla	ADC	QDA	0.530 ± 0.029	0.486 ± 0.041	0.506 ± 0.028	0.476 ± 0.031	0.643 ± 0.021
		RF	0.597 ± 0.028	0.585 ± 0.042	0.570 ± 0.028	0.557 ± 0.030	0.738 ± 0.015
	D	QDA	0.478 ± 0.023	0.469 ± 0.025	0.460 ± 0.023	0.451 ± 0.023	0.629 ± 0.013
		RF	0.386 ± 0.037	0.334 ± 0.048	0.359 ± 0.034	0.333 ± 0.033	0.553 ± 0.025
	D*	QDA	0.483 ± 0.027	0.478 ± 0.030	0.468 ± 0.027	0.459 ± 0.026	0.648 ± 0.017
		RF	0.465 ± 0.035	0.399 ± 0.050	0.434 ± 0.035	0.405 ± 0.036	0.601 ± 0.026
	f	QDA	0.524 ± 0.029	0.523 ± 0.028	0.518 ± 0.028	0.518 ± 0.028	0.690 ± 0.017
		RF	0.605 ± 0.027	0.594 ± 0.031	0.589 ± 0.028	0.585 ± 0.031	0.744 ± 0.012
Left renal medulla	ADC	QDA	0.584 ± 0.033	0.545 ± 0.045	0.556 ± 0.033	0.532 ± 0.035	0.693 ± 0.019
		RF	0.525 ± 0.033	0.493 ± 0.044	0.501 ± 0.035	0.488 ± 0.040	0.672 ± 0.017
	D	QDA	0.602 ± 0.029	0.590 ± 0.038	0.584 ± 0.029	0.570 ± 0.029	0.745 ± 0.013
		RF	0.504 ± 0.026	0.474 ± 0.043	0.479 ± 0.026	0.457 ± 0.030	0.666 ± 0.019
	D*	QDA	0.289 ± 0.030	0.301 ± 0.095	0.298 ± 0.032	0.242 ± 0.029	0.478 ± 0.024
		RF	0.495 ± 0.028	0.479 ± 0.037	0.474 ± 0.028	0.462 ± 0.031	0.653 ± 0.018
	f	QDA	0.513 ± 0.026	0.514 ± 0.027	0.501 ± 0.026	0.501 ± 0.026	0.729 ± 0.017
		RF	0.602 ± 0.028	0.588 ± 0.036	0.579 ± 0.028	0.568 ± 0.030	0.764 ± 0.014
Right renal cortex	ADC	QDA	0.550 ± 0.023	0.528 ± 0.031	0.517 ± 0.022	0.497 ± 0.024	0.640 ± 0.019
		RF	0.447 ± 0.029	0.431 ± 0.092	0.417 ± 0.028	0.381 ± 0.033	0.552 ± 0.022
	D	QDA	0.554 ± 0.032	0.546 ± 0.037	0.541 ± 0.030	0.536 ± 0.031	0.687 ± 0.018
		RF	0.486 ± 0.030	0.428 ± 0.054	0.461 ± 0.029	0.424 ± 0.034	0.664 ± 0.020
	D*	QDA	0.480 ± 0.026	0.467 ± 0.031	0.462 ± 0.026	0.450 ± 0.027	0.626 ± 0.017
		RF	0.479 ± 0.024	0.483 ± 0.026	0.467 ± 0.024	0.467 ± 0.024	0.590 ± 0.016
	f	QDA	0.372 ± 0.041	0.360 ± 0.044	0.363 ± 0.041	0.357 ± 0.042	0.586 ± 0.021
		RF	0.471 ± 0.033	0.464 ± 0.038	0.457 ± 0.035	0.455 ± 0.037	0.625 ± 0.016
Left renal cortex	ADC	QDA	0.555 ± 0.033	0.482 ± 0.042	0.521 ± 0.033	0.492 ± 0.033	0.666 ± 0.014
		RF	0.561 ± 0.027	0.521 ± 0.051	0.530 ± 0.028	0.503 ± 0.036	0.680 ± 0.017
	D	QDA	0.519 ± 0.024	0.451 ± 0.030	0.487 ± 0.024	0.461 ± 0.024	0.658 ± 0.016
		RF	0.393 ± 0.034	0.360 ± 0.053	0.364 ± 0.033	0.336 ± 0.035	0.535 ± 0.031
	D*	QDA	0.467 ± 0.026	0.463 ± 0.028	0.448 ± 0.025	0.444 ± 0.025	0.572 ± 0.017
		RF	0.498 ± 0.034	0.482 ± 0.046	0.478 ± 0.036	0.468 ± 0.040	0.691 ± 0.019
	f	QDA	0.501 ± 0.024	0.484 ± 0.030	0.479 ± 0.024	0.460 ± 0.026	0.614 ± 0.015
		RF	0.468 ± 0.035	0.446 ± 0.041	0.450 ± 0.037	0.443 ± 0.039	0.637 ± 0.023

The classification model using TFs derived from the ipsilateral kidney and renal medulla/cortex showed lower performance than the model using TFs derived from both kidneys. There were no significant differences between TFs derived from the renal medulla and the cortex.

## Discussion

This study aimed to determine whether combining TA with IVIM-derived diffusion and perfusion metrics would improve the accuracy of MRI-based assessments of renal severity in patients with CKD. While the TA models derived from IVIM parameters showed limited incremental benefits over conventional ADC, the ADC-based model using a QDA classifier demonstrated good discriminatory performance, achieving AUC values exceeding 0.80. Therefore, the hypothesis that the biexponential model would provide superior diagnostic capabilities compared to the monoexponential model was not supported by our findings. A sub-analysis separately evaluating the renal cortex and medulla also failed to improve diagnostic performance or demonstrate a significant advantage.

Our findings regarding the utility of DWI and IVIM are consistent with those of a large body of existing literature. Numerous studies have demonstrated a relationship between DWI, particularly the ADC derived from the monoexponential model, and renal impairment. These studies have consistently shown associations among ADC values, CKD severity, and eGFR [24-27]. Thus, the ability of ADC to reflect overall renal function is well established.

The literature on the biexponential model (IVIM) in renal imaging is less conclusive, with varying reports on its superiority over the monoexponential model. Several studies have explored the relationship between renal dysfunction or eGFR and IVIM parameters such as f and D [24-27]. Some researchers have reported the potential of the biexponential model to outperform the monoexponential model. For example, one study histopathologically evaluated renal fibrosis and correlated the parameters of each model with those of glomerular and tubulointerstitial fibrosis [28]. Although ADC showed no significant correlation with either component, D demonstrated a moderately negative correlation [28].

However, contrasting results have been reported previously. Other studies have reported no significant differences in performance between the monoexponential and biexponential models [29]. In a study focusing on IgA nephropathy, the histopathological assessment of renal fibrosis showed the strongest correlation with f, followed by D*, ADC, and D in descending order [30]. These findings indicate that a consensus regarding model superiority is yet to be reached. This lack of consensus underscores the need for further investigations into the optimal DWI model for renal assessment. In our study, the IVIM-derived parameters (D, D*, and f) positively correlated with the severity of renal impairment, with correlation coefficients exceeding 0.7, which is consistent with the results of many previous studies.

Our study investigated the utility of TA in DWI. Previous studies have shown an association between TFs extracted from DWI and the severity of renal impairment [20,31]. Furthermore, a study incorporating ML techniques reported similar associations [19]. Our results, which demonstrate the potential of TA, are consistent with those of prior reports. Certain original first-order features identified as potentially useful in our analysis, such as Entropy and Skewness, overlap with those reported previously [20]. Additionally, three other features, namely, Energy, Entropy, and Correlation, are consistent with those highlighted in a previous study [19].

Interestingly, contrary to several previous studies that demonstrated that the renal cortex reflects renal impairment more sensitively than the medulla [31,32], our study did not reveal any significant differences between these regions. This discrepancy from earlier findings may be attributable to factors such as limited image quality and a small sample size. To the best of our knowledge, we additionally explored relationship between each imaging parameter and renal impairment by separately analyzing bilateral and unilateral kidneys.

The present study had several limitations. First, because the images were acquired between 2008 and 2011 using an older 1.5-Tesla MRI system compared with current scanners, MRI image quality was suboptimal, which made accurate assessment challenging. Second, the small sample size may have limited the robustness of the findings. Third, this study did not include healthy volunteers, thus preventing a comparison across the entire spectrum of patients with CKD with a normal control group. Fourth, the histopathological correlations were not examined. Previous studies have shown an association between TFs and hepatic fibrosis. To the best of our knowledge, no study has clarified the relationship between TFs and renal fibrosis. These issues should be addressed in future studies.

An important implication of this study is that theoretically advanced diffusion models do not necessarily translate into superior performance in real-world clinical datasets. Although IVIM enables separate estimations of the diffusion and perfusion components, its parameters, particularly D*, are sensitive to noise, motion, and fitting instability [33]. In contrast, ADC represents a more robust and reproducible metric derived from a simpler model. TA may further amplify these differences because it relies on stable spatial intensity patterns rather than subtle physiological signals. From this perspective, the superior performance of ADC-based TA observed in this study may reflect the practical robustness rather than the methodological inferiority of IVIM.

## Conclusions

In conclusion, although the integration of these advanced imaging techniques showed promise, and also this study is proof-of-concept and internally validated, the TA model derived from ADC maps using a QDA classifier demonstrated superior accuracy compared to models based on IVIM parameters. Consequently, the utility of TA using ADC maps was reaffirmed. Although the overall performance was not optimal, the findings demonstrated reproducible and clinically interpretable discrimination of the severity of renal dysfunction. Future research should prioritize larger multicenter cohorts, enhanced MRI acquisition protocols to improve image quality and resolution, more external tests, standardized IVIM fitting pipelines, 3D/whole-kidney segmentation to improve generalizability and validation against histopathological findings to definitively establish the clinical utility of TA in the non-invasive assessment of the severity of renal dysfunction.
